# Improving GP registration and access for migrant health

**DOI:** 10.3399/bjgp22X718301

**Published:** 2022-01-28

**Authors:** Yusuf Ciftci, David N Blane

**Affiliations:** Doctors of the World UK, London.; General Practice and Primary Care, Institute of Health and Wellbeing, University of Glasgow, Glasgow.

## INTRODUCTION

Migrants to the UK — including refugees, asylum seekers, and undocumented migrants — experience health inequities due to social exclusion, discrimination, language barriers, and, for some, restricted entitlement to health care due to their immigration status.^1^ In this article we echo recent calls for more inclusive migrant health care^2^ and consider the impacts of COVID-19 on access to primary care for undocumented migrants in particular.

## MIGRANTS IN VULNERABLE CIRCUMSTANCES IN THE UK

A recent report estimated that there are 674 000 undocumented migrants across the UK, with 397 000 in London.^3^ The number of undocumented children in the UK increased by 79% between 2011 and 2017, and almost half were born in the UK.^3^ Home Office statistics show that, every year, approximately 30 000 asylum applications are lodged and roughly 20 000 of these are granted asylum, alternative forms of leave, or resettlement.^4^ These figures show that there are many migrants who do not have a secure immigration status, right to work, or access to welfare services leaving them with inadequate housing and potential destitution. These vulnerable situations make it difficult for migrants to access the healthcare services they need, despite many having additional healthcare needs (see Box 1).

**Box 1.** Additional health needs of migrants

Migrant populations are diverse but many face a ‘triple burden’ of non-communicable diseases infectious diseases and mental health problems due to a range of adverse experiences before during and after migration.^5^ **Non-communicable diseases:** The World Health Organisation (WHO) reports that refugees and migrants have a higher incidence prevalence and mortality rate for chronic conditions such as diabetes and that cancer is more likely to be diagnosed at an advanced stage in refugees and migrants.^6^ A UK report also reported poorer physical health outcomes due to experiences during and after migrating to the UK.^7^ **Infectious diseases:** Some infectious diseases are more common in other countries due to different health systems and vaccination programmes. Also living in overcrowded conditions with poor sanitation before during and after migration increases risks of bacterial viral and parasitic infections. The UN programme on HIV and AIDS recognises that displacement and migration can place people in situations of heightened vulnerability to HIV. **Mental health:** People seeking asylum are more likely to experience mental health problems than the general population including post-traumatic stress disorder and longer-term effects of inhumane treatment such as torture and sexual violence.^8^ Depression and anxiety are also commonly reported linked to lengthy asylum-seeking processes and poor socioeconomic conditions such as unemployment or isolation.^9^

## IMPACT OF COVID-19

The COVID-19 pandemic has highlighted the need for a more inclusive approach in the way health services are provided. A recent systematic review found that migrants are at increased risk of contracting COVID-19.^10^ A detailed analysis by Doctors of the World (DOTW) of the early impacts of the pandemic on excluded people in England showed that COVID-19 created new destitution and homelessness, particularly among those with no recourse to public funds.^11^ The report revealed that de-registration of patients who have been temporarily housed outside their catchment areas or who are socially distancing/isolating at an alternative address affected many marginalised people’s healthcare access to (and trust in) health services and created delays in access to medications such as antidepressants, creating additional barriers to care.^11^

During the pandemic, the DOTW clinics witnessed the vulnerable circumstances many migrants have been living in and the ongoing barriers to accessing primary care. While 44% of patients who attended DOTW clinics during the 3-month period prior to the first lockdown stated that they have inadequate or insecure housing, this figure increased to 60% in the 3-month period starting with the first lockdown, showing the impact on housing.^12^ Furthermore, of the 927 patients attending during 2020, 407 (44%) reported that they could not access health care due to ongoing barriers. Of these, 258 (28% of total) stated that they lack understanding or knowledge of their health rights and the healthcare system in the UK, 103 (11%) faced administrative barriers such as inability to show proof of ID or address, and 73 (8%) declared that they feared arrest or immigration enforcement in the healthcare services. The figures were all increases from previous years’ statistics (unpublished data, DOTW, available from authors on request).

The reconfiguration of primary care towards ‘remote-by-default’ consulting,^13^ primarily by telephone, represents a further potential barrier to accessing care for migrant populations. The DOTW report found that many migrants lack access and skills to use technology or are unable to pay for access to broadband or mobile data.^11^ The reduced physical access to surgeries has also resulted in reduced support with registration, attendance, and signposting to other services, alongside challenges with communication and identifying safeguarding issues.^14^

## THE ROLE OF PRIMARY CARE

General practice has played a key role in efforts to tackle health inequities among migrant populations. Primary care policy is clear that in England, Scotland, and Wales, immigration status should not affect entitlement to register for and receive primary care services. The NHS England *Primary Medical Care Policy and Guidance Manual* clearly states that lack of proof of identity or address *‘would not be considered reasonable grounds to refuse to register a patient.’* ^15^ This is encouraging, but further actions need to be taken to ensure the guidance is implemented. Similarly, NHS Scotland and NHS Wales should issue clear and accessible guidance and effective communication to GP practices to ensure inclusive access to migrants and other marginalised communities.

More recently, NHS England launched a new GP registration campaign highlighting that *‘everyone is welcome in General Practice’*, accompanied by resources for patients to show their entitlements when they present at general practice for registration.^16^ This could play a role in addressing the pandemic too, as GP registration is one of the most effective ways of enabling access to the COVID-19 vaccine. However, inclusive policy often has a limited impact on the ground due to discrimination and social exclusion of migrants.^17^ The UK government’s new Nationality and Immigration Bill, which proposes to penalise irregular entry to the UK asylum protection system, is likely to exacerbate barriers to access primary care.^18^

Examples of good inclusive registration practice should be recognised and shared widely. DOTW’s Safe Surgeries Initiative is an innovative evidence-based programme that promotes a ‘community of practice’ model to tackle barriers to primary care and health inequalities faced by migrants.^19^ More than 600 general practices have signed up to the Initiative,^18^ which includes free training and access to expert advice on migrants’ entitlement to health care, resources like translated posters, and a toolkit that helps address administrative and trust barriers.^20^ Finally, patient advocacy resources, similar to NHS England’s GP Access cards,^21^ can help migrant groups know their rights and entitlements to health care, providing practical guidance on how to access services.

## CONCLUSION

Many migrants — and undocumented migrants in particular — already face barriers to accessing primary care and are at increased risk of digital exclusion too. We join others in calling for targeted additional support to improve access to services, and engagement with local migrant community groups to provide clear, concise, and language-specific written and non-written resources.^14^ This will be important to facilitate COVID-19 vaccine uptake but is also critical for inclusive migrant health.
